# A Multi-Channel Method for Retrieving Surface Temperature for High-Emissivity Surfaces from Hyperspectral Thermal Infrared Images

**DOI:** 10.3390/s150613406

**Published:** 2015-06-08

**Authors:** Xinke Zhong, Jelila Labed, Guoqing Zhou, Kun Shao, Zhao-Liang Li

**Affiliations:** 1 ICube, UdS, CNRS, 300 Bld Sebastien Brant, CS10413, 67412 Illkirch, France; E-Mails: x.zhong@unistra.fr (X.Z.); labed@unistra.fr (J.L.); 2 Guangxi Key Laboratory of Spatial Information and Geomatics, Guilin University of Technology, Guilin 541004, China; E-Mail: gzhou@glut.edu.cn; 3 School of Computer and Information, Hefei University of Technology, Hefei 230009, China; E-Mail: shaokun@hfut.edu.cn; 4 Key Laboratory of Agri-informatics, Ministry of Agriculture/Institute of Agricultural Resources and Regional Planning, Chinese Academy of Agricultural Sciences, Beijing 100081, China

**Keywords:** surface temperature retrieval, spectral information, hyperspectral thermal infrared images, IASI

## Abstract

The surface temperature (ST) of high-emissivity surfaces is an important parameter in climate systems. The empirical methods for retrieving ST for high-emissivity surfaces from hyperspectral thermal infrared (HypTIR) images require spectrally continuous channel data. This paper aims to develop a multi-channel method for retrieving ST for high-emissivity surfaces from space-borne HypTIR data. With an assumption of land surface emissivity (LSE) of 1, ST is proposed as a function of 10 brightness temperatures measured at the top of atmosphere by a radiometer having a spectral interval of 800–1200 cm^−1^ and a spectral sampling frequency of 0.25 cm^−1^. We have analyzed the sensitivity of the proposed method to spectral sampling frequency and instrumental noise, and evaluated the proposed method using satellite data. The results indicated that the parameters in the developed function are dependent on the spectral sampling frequency and that ST of high-emissivity surfaces can be accurately retrieved by the proposed method if appropriate values are used for each spectral sampling frequency. The results also showed that the accuracy of the retrieved ST is of the order of magnitude of the instrumental noise and that the root mean square error (RMSE) of the ST retrieved from satellite data is 0.43 K in comparison with the AVHRR SST product.

## 1. Introduction

The surface temperature (ST) of surfaces with high emissivity, e.g., dense vegetation and sea surfaces, is a key parameter in climate systems. Sea surface temperature (SST) retrieval is applied in thematic areas such as numerical weather/climate forecasting [1], and global climate change monitoring [2]. Surface temperature of high-emissivity surfaces in land is required for atmospheric correction of thermal infrared image [3]. Thermal infrared remote sensing has become an effective approach for measuring ST for high emissivity surfaces on a larger spatial scale [4,5]. We use the narrow definition of surface temperature, where surface temperature is defined as the temperature of the surface at radiative equilibrium.

The ST of high-emissivity surfaces can be retrieved from satellite-based multispectral thermal infrared data by nine methods: the single-channel method [6], the split-window (SW) method [7] and the multi-channel method [8,9,10], the multi-angle method [11], the physical-based day/night operational method [12], the temperature and emissivity separation (TES) method [13], the multi-temporal physical method [14], the Kalman filter physical method [15] and the two-step retrieval method (TSRM) [16,17]. The single-channel method requires an accurate atmospheric profile. This condition is difficult or even impossible to satisfy in most practical situations. The split-window method utilizes differential atmospheric absorptions in two adjacent channels centred at 11 μm and 12 μm, which does not require information about the atmospheric profile for ocean applications at the time of the acquisition [7]. However, the SW method requires accurate atmospheric water vapour content [3]. The multi-channel method uses characteristics of the mid-infrared (MIR 3–6 µm) channel *i*_1_ at 3.9 μm and channel *i*_2_ centred at 8.7 µm to improve atmospheric correction at night, which does not require atmospheric water vapour content [8,10]. However, solar radiance at the MIR channel limits the use of the multi-channel method. Similar to the principle of the SW method, the multi-angle method is based on the differential water vapour absorption measured by sensor from different angles. The multi-angle method suffers from ST angular dependence [18]. The physical-based day/night operational method utilizes two-time measurements at 7 MIR and thermal infrared (TIR) channels to constrain the ill-posed temperature/emissivity separation with known atmospheric corrections [17]. However, the physical-based day/night operational method suffers from problems of geometry mis-registration, variations in the viewing zenith angle and inaccurate atmospheric correction [19]. The TES method relies on an empirical relationship between spectral contrast and minimum emissivity to separate land surface temperature (LST) and land surface emissivity (LSE) from five atmospherically corrected Advanced Spaceborne Thermal Emission and Reflection Radiometer (ASTER) TIR data [13]. However, the TES method exhibits significant errors under hot and wet atmospheric conditions [20]. The multi-temporal physical method [14] and the Kalman filter physical method [15] utilize the invariance feature of LSE measured within a short time period (six hours) to separate LST and LSE from geostationary thermal infrared radiances provided that good atmospheric correction has been perform. The TRSM method simultaneously retrieves the atmospheric profiles, LST and LSE from the MODIS channel data, which does not require atmospheric correction [17]. However, the requirement of adequate channels and the TRSM method’s complex nature make it difficult to apply.

The methods mentioned above use satellite data measured at several broad channels, therefore they cannot be applied to hyperspectral thermal infrared (HypTIR) data. The HypTIR data with thousands of channels provide plenty of information on the atmosphere and the observed surface. Therefore, new methods are required for retrieving ST for high-emissivity surfaces from HypTIR satellite data.

Currently, there is large amount of HypTIR data acquired by space-borne sensors. The earliest sensor, Interferometric Monitor for Greenhouse Gases (IMG), was launched in 1996, but it failed in 1997. The first successful sensor, the Atmospheric InfraRed Sounder (AIRS) [21], was launched in 2002 and has been providing HypTIR data ever since. There are HyperTIR data observed by other space-borne sensors, such as the Infrared Atmospheric Sounding Interferometer (IASI) [22] and the Cross-track Infrared Sounder (CrIS) [23]. In the future, the infrared sounder (IRS) [24] will also provide this type of HypeTIR data. There is a pressing need for methodological development in order to retrieve ST from these space-borne HypTIR data.

There are mainly five types of methods for retrieving ST for high-emissivity surfaces from space-borne HypTIR data: the principal component regression method [25,26,27,28,29], the artificial neural network (ANN) method [30,31], the stepwise LST and LSE retrieval method [32], the simultaneous LST and LSE retrieval method [33], the physical simultaneous atmospheric profiles, LST and LSE retrieval method [34,35,36,37]. The ANN method and principal component regression method are based on a linear/nonlinear empirical relationship between principal component amplitudes of brightness temperature spectrum at TOA and ST. The principal component regression method and the ANN method do not require extra atmospheric data and are fast enough for near real-time application [28]. However, the principal component regression method and the ANN method require thousands of channels and have much error for complex physical situations [33]. For example, aerosols contaminate the principal component regression method and the ANN method. The stepwise LST and LSE retrieval method relies on the phenomenon that LSE is close to unity at a certain channel to separate LST and LSE with known atmospheric profile. The stepwise LST and LSE retrieval method requires accurate atmospheric profile. The simultaneous LST and LSE retrieval method depends on an empirical relationship between principal components of LSE and channel LSEs to constraint iterative solution of LSE and LST with known atmospheric profile. The simultaneous LST and LSE retrieval method also requires accurate atmospheric profile. The physical simultaneous atmospheric profile, LST and LSE retrieval method utilizes physical constraint based on spectral smoothness characteristic of LSE to iterative solve LST, LSE and atmospheric profile simultaneously with the support of atmospheric radiative transfer model. The physical simultaneous retrieval method does not require atmospheric profile, but it has low computation efficiency because of its complex nature [31]. The objective of this paper is to develop a multi-channel method for retrieving ST for high-emissivity surfaces from space-borne HypTIR images.

The proposed method is suitable for retrieving ST for high-emissivity surfaces from HypTIR measurements containing damaged data and provides flexibility for retrieving ST for high-emissivity surfaces with taking the complex physical situations mentioned above into account in future. The proposed method can be used for near real time production of ST for high-emissivity surfaces from space-borne HypTIR data and for providing first-guess value of ST for the physical method.

This paper is organized as follows: Section 2 presents the proposed method. The evaluation of the proposed method is given in Section 3. The sensitivity of the proposed method to spectral sampling frequency and instrumental noise is shown in Section 4. The application of the proposed method to satellite data is shown in Section 5, and the last section provides the conclusion.

## 2. Methodological Development

### 2.1. Physical Base of the Method

Assuming that the land surface is a black body, the radiance L(λ*_i_*) at TOA at a HypTIR channel can be written as:

(1) $${\text{L(λ}}_{i}\text{)=B(}{\text{T}}_{s},{\text{λ}}_{i}{\text{)τ(λ}}_{i}{\text{)+L}}_{P}{\text{(λ}}_{i}\text{)}$$

where B(T*_s_*, λ*_i_*) is the surface radiance at a central wavelength λ*_i_* of channel *i* with a surface temperature of T*_s_*, L*_p_* is the upwelling radiance emitted by the atmosphere, and τ is the atmospheric transmittance.

If Equation (1) is linearized around ST and the wavelength λ*_i_* in this equation is omitted, Equation (1) can be rewritten as:

(2) $${\text{Tb}}_{i}\text{= }{\text{τ}}_{i}{\text{T}}_{s}\text{+ (1}-{\text{τ}}_{i}{\text{)Ta}}_{i},\;1\leq i\leq p$$

where Tb*_i_* is the brightness temperature at TOA at channel *i*, Ta*_i_* is the equivalent atmospheric temperature at channel *i*, τ*_i_* is the transmittance at channel *i*, and p is the number of channels selected for retrieving ST.

Inspired by the split-window method for ST retrieval, we propose a multi-channel method for retrieving ST for high-emissivity surfaces from HypTIR data. In this method, ST can be written as:

(3) $$T_{s}=w_{0}+\sum_{i=1:\text{p}}w_{i}Tb_{i}$$

where *w_i_* are regression coefficients. The number of channels is p, and the centre wavenumbers at channel *i* (*i* = [1,p]), and coefficients *w_i_* (*i* = [0,p]) can be determined using stepwise regression with simulation data.

### 2.2. Data for Simulation

Although there are large amounts of HypTIR data measured at TOA, it is still difficult to find spatially and temporally collocated atmospheric moisture and temperature profile data. Additionally, there are few field-measured ST data at the spatial scale of a satellite FOV (12 km for IASI). Therefore, we have resorted to simulation data for determining the parameters in Equation (3). Specifically, we have used typical atmospheric profile data and typical ST data to create simulation data for determining the parameters in Equation (3).

**Figure 1:**
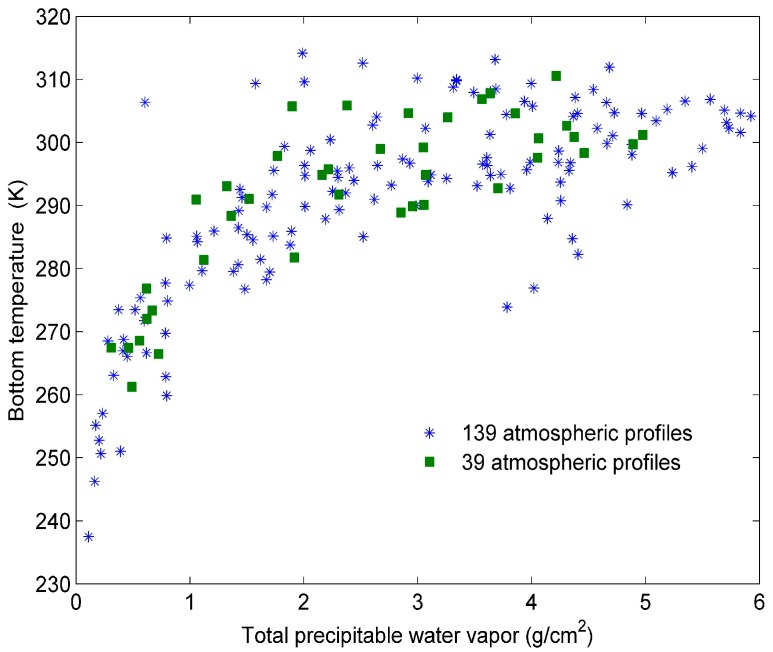
The bottom temperatures as a function of the total precipitable water vapour for the 139 atmospheric profiles and other 39 atmospheric profiles.

We have selected typical profiles from the Thermodynamic Initial Guess Retrieval (TIGR) database for simulation [38,39] in two steps. First, we have classified the 2311 clear-sky TIGR profiles into six groups according the concentration of water vapour. The total precipitable water-vapour ranges of the six groups are between 0 and 1 g/cm^2^, between 1 and 2 g/cm^2^, between 2 and 3 g/cm^2^, between 3 and 4 g/cm^2^, between 4 to 5 g/cm^2^, and between 5 and 6 g/cm^2^, respectively. After that, we have randomly selected nearly 23 profiles from each group to make sure the selected profiles were representative. Each atmospheric profile has 40 layers from 1013 hPa to 0.05 hPa. The air mass types for the selected atmospheric profiles are tropical, temperate, cold temperate and summer polar, cold polar, and winter polar types. The total precipitable water vapours of the selected atmospheric profiles range from 0 g/cm^2^ to 6 g/cm^2^. The variation of bottom temperature with the total precipitable water vapour for the 139 atmospheric profiles is presented in Figure 1. The method for determining clear-sky atmospheric profiles from the TIGR database has been detailed by [40].

To describe the rapid variation of LST for each profile, the six STs for simulation are sums of bottom atmospheric temperature (Ta_0_) and one out of six perturbations. The six perturbations are [−15 K, −5 K, 0 K, 5 K, 10 K, and 15 K] when Ta_0_ < 280 K, and these perturbations are [−10 K, −5 K, 0 K, 5 K, 10 K, and 20 K] when Ta_0_ ≥ 280 K [30].

### 2.3. Determination of Centre Wavenumbers of Channel i (i = [1,p]) and Coefficients wi (i = [0,p])

**Figure 2:**
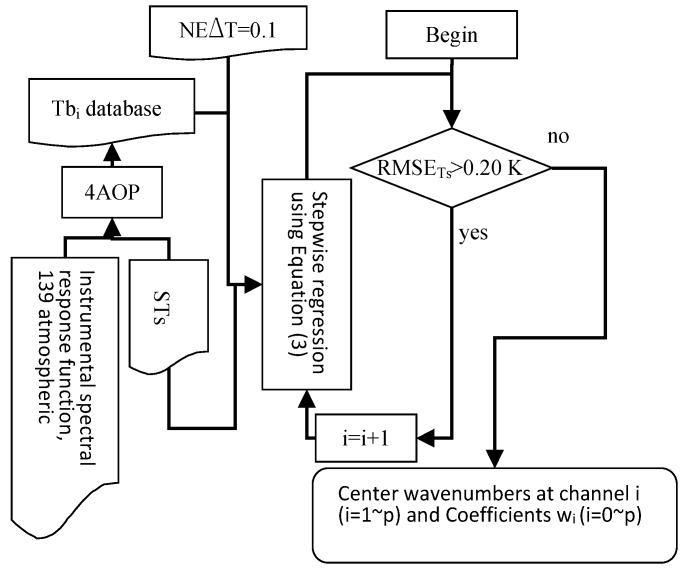
The scheme for determining the centre wavenumbers of channel *i* (*i* = [1,p]) and the coefficients *w_i_* (*i* = [0,p]).

We have used the stepwise regression method with the simulation data to determine the centre wavenumbers at channel *i* and coefficients *w_i_* in Equation (3) for IASI. The procedure is shown in Figure 2.

For this study, we have simulated large IASI data using Operational release for Automatized Atmospheric Absorption Atlas (4A/OP) [41,42] using the data mentioned in Section 2.2. The 4A/OP is used to simulate atmospheric transmittance and upward radiance using atmospheric profile data. Brightness temperature database is calculated using Equation (1) with output atmospheric radiative terms of 4A/OP. The spectral interval and spectral sampling frequency for simulation are 800–1200 cm^−1^ and 0.25 cm^−1^, respectively. The viewing angle for simulation is restricted to nadir observation. For each simulation case, a random noise with dimension of 1601 generated by a Matlab random number generator with NE∆T of 0.1 K is added. The noise equivalent temperature difference (NE∆T) has been set according to that of the IASI [43]. Because O_3_ has a strong absorption feature in HypTIR radiance spectrum, only HypTIR data at channels in the spectral interval of 800–985 cm^−1^ and in the spectral interval of 1150–1200 cm^−1^ have been used for stepwise regression [44].

The stepwise regression is used to determine the centre wavenumbers of channel *i* and the coefficients *w_i_* with the simulation data above. First, the channel centred at a wavenumber of 1158.5 cm^−1^, where transmittance is the largest, is selected as the initial channel.

In step *m* + 1 of the stepwise regression, for each remaining channel, a linear relationship fitted by the least square method is written as:

(4) $$T_{s}=w_{0}+\sum_{i=1}^{m}w_{i}Tb_{i}+w_{m+1}Tb_{m+1}$$

where Tb*_i_* (*i* = [1,m]) are the channel brightness temperatures determined before this step, and Tb*_m_*_+1_ is the brightness temperature at each remaining channel. The coefficients *w_i_* (*i* = [1,m + 2]) is calculated by the following equation:

(5) $$W={\left(X^{T}X\right)}^{-1}X^{T}Y$$

where W is the coefficient vector of dimension *m* + 2, X is HypTIR brightness temperature *n* × (*m* + 2) matrix, and Y is the LST vector of dimension n. Here, X contains n samples of the *m* + 1 channel HypTIR brightness temperatures. A sum of squares for partial regression (U*_k_*) is used to calculate the contribution of Tb*_m_*_+1_ and is defined as:

(6) $$U_{k}=SS_{k}-SS_{-k}$$

where SS*_k_* and SS*_−k_* are the sum of the squares for regression with channel *k*, and without channel *k*, respectively. The sum of squares for regression is defined as:

(7) $$SS={\sum_{i=1}^{n}\left(w_{0}+\sum_{j=1}^{l}w_{0}Tb_{ij}-\bar{y}\right)}^{2}$$

where *l* is the number of channels and *n* is the number of simulation cases, and *ȳ* is the mean of *n* samples of LSTs. The (*m* + 1)-th channel HypTIR brightness temperature added by the stepwise regression is the one with largest partial regression square sums among the remaining channel HypTIR brightness temperatures. We also check to see if the spectral interval of two nearby central wavenumbers is larger than 4.5 cm^−1^. If not, HypTIR brightness temperature of this channel is replaced by the one with second-largest sum of squares for partial regression.

The criterion for determining the number of channels for Equation (3) is that the root mean square error (RMSE) of the ST retrieved using Equation (3) from the simulation data mentioned above is less than 0.2 K. The output coefficients *w_i_* and centre wavenumbers of channel *i* are the solutions.

**Figure 3:**
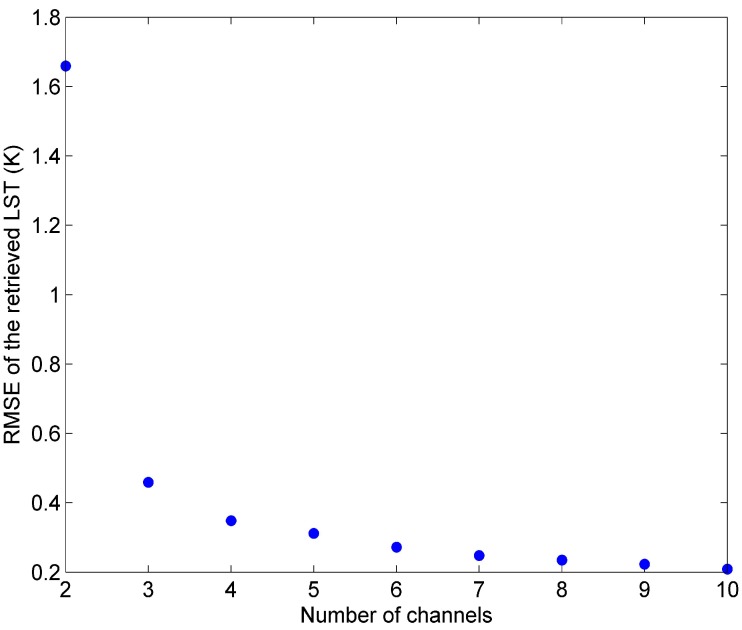
The variation of RMSE of the retrieved ST with the number of channels in the process for determination of centre wavenumbers and coefficients.

The variation of RMSE of the retrieved ST with the number of channels in the process of determining the centre wavenumbers of channel *i* and the coefficients *w_i_* is shown in Figure 3: the error of the ST that is retrieved using the corresponding regression equation with the simulation data above decreases with the growing number of channels, and the RMSE of the retrieved ST is less than 0.2 K when the number of channels is larger than 10.

**Figure 4:**
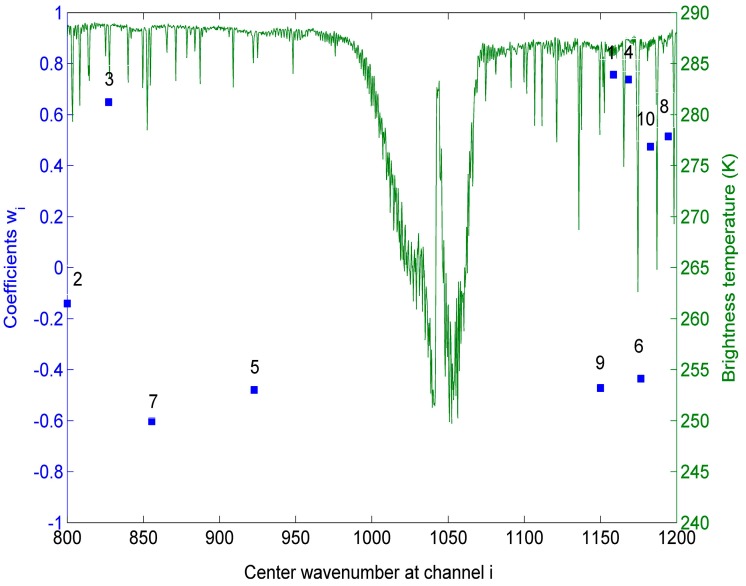
The centre wavenumbers at channel *i* (*i* = [1,p]) and coefficients *w_i_* (*i* = [0,p]) superimposed on a typical IASI spectrum. (w_0_ = 2.486; the No. above each blue square indicate the order of each channel in the determination process).

The centre wavenumbers of channel *i* (*i* = [1,p]) and the coefficients *w_i_* are shown in Figure 4. From this figure, we can see that the centre wavenumbers correspond to the wavenumbers where water vapour absorption is weak. The reason may be that the assumption that ST can be expressed as a linear function of p HypTIR brightness temperatures is more reliable at these wavenumbers. The Figure 4 also shows that all the coefficients *w_i_* are varying in a relatively small range between: −0.80~0.80.

## 3. Evaluation of the Method

**Figure 5:**
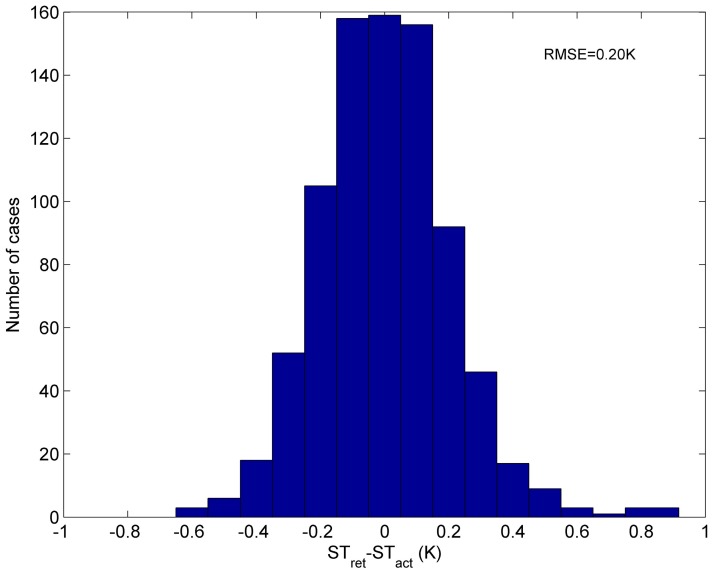
Error of the ST retrieved by Equation (3) from simulation data with the 139 atmospheric profiles. (ST_ret_ = the retrieved ST, ST_act_ = the true ST).

First, we have evaluated the developed method by comparing the ST retrieved by that developed method from simulation data in Section 2 with the true input ST. The error of the retrieved ST is shown in Figure 5. From this figure, we can see that the RMSE of the retrieved ST is approximately 0.20 K, and the error of the retrieved ST ranges from −0.6 to 0.9 K. Consequently, ST can be accurately retrieved using Equation (3), with only 10 measurements in the spectral interval of 800–1200 cm^−1^.

To evaluate the method independently, we have selected 39 other profiles from the clear-sky TIGR database as mentioned in Section 2.2. We have classified all the clear-sky TIGR profiles into five groups, except for the 139 profiles mentioned in Section 2.2, and selected nearly eight profiles from each of these five groups for evaluation. The total precipitable water-vapour ranges of the five groups are between 0 and 1 g/cm^2^, between 1 and 2 g/cm^2^, between 2 and 3 g/cm^2^, between 3 and 4 g/cm^2^ and between 4 and 5 g/cm, respectively. The variation of the bottom temperature with total precipitable water vapour for the 39 atmospheric profiles is represented in Figure 1 by green squares. For each atmospheric profile, the five STs for independent simulation are the sums of bottom temperature with one of five temperature perturbations. The five temperature perturbations and other parameters for this independent simulation are the same as those in Section 2.

**Figure 6:**
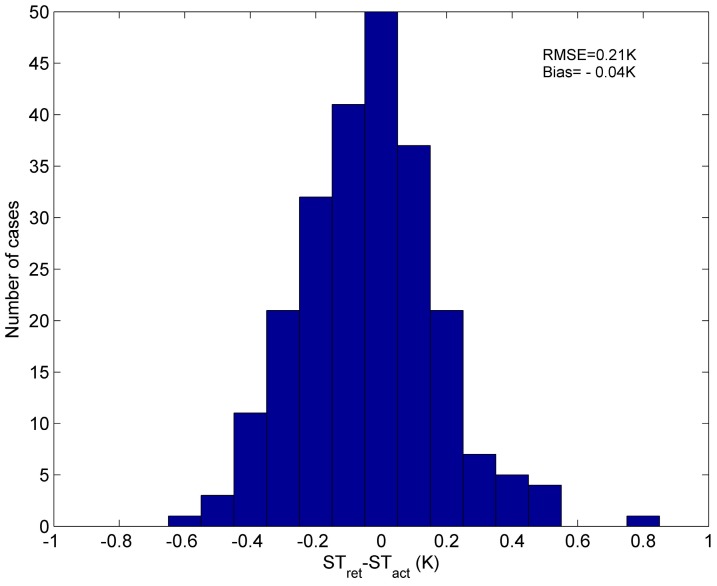
Error of the ST retrieved by Equation (3) from independent simulation data with the 39 atmospheric profiles. (ST_ret_ = the retrieved ST, ST_act_ = the true ST).

The error of ST retrieved from this independent simulation data is shown in Figure 6. The RMSE of ST retrieved by Equation (3) is 0.21 K. Our method is quite accurate and promising.

## 4. Sensitivity Analysis of the Method

### 4.1. Sensitivity to Spectral Sampling Frequency

We have analysed the sensitivity of the method to spectral sampling frequency by refitting the coefficients *w_i_* in Equation (3) for each spectral sampling frequency, using five simulation databases and studying the error of ST retrieved using these refitted coefficients *w_i_* from five other independent simulation databases.

**Figure 7:**
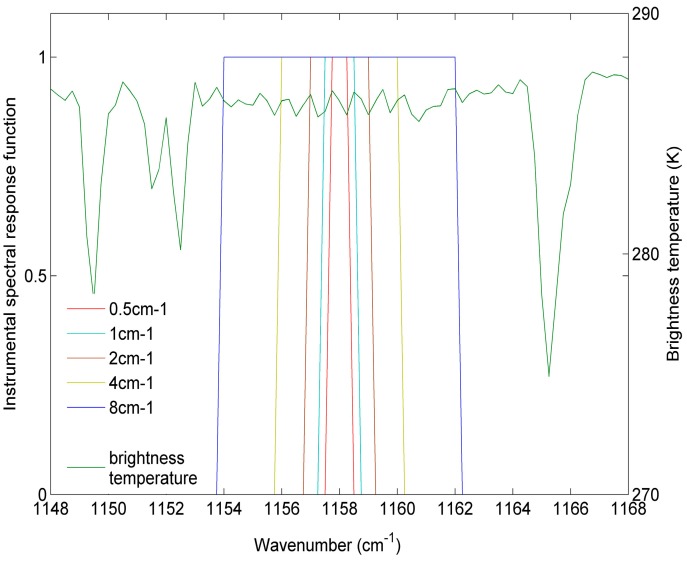
The five ISRFs at the channel with the centre wavenumber = 1158 cm^−1^ superimposed on one typical IASI spectrum in the spectral interval of 1148–1168 cm^−1^.

To refit the coefficients *w_i_*, we have created five simulation databases for five HypTIR sensors with spectral sampling frequencies = 0.5, 1, 2, 4, 8 cm^−1^. We assumed that the five sensors have 10 channels with the centre wavenumbers shown in Figure 4. The instrumental spectral response functions (ISRFs) for the five sensors are rectangular impulse functions. The ISRFs for the five sensors at one channel are shown in Figure 7. The simulation database for each of the five sensors is resampled from the simulation data mentioned in Section 2 using each ISRF. The coefficients *w_i_* refitted for each spectral sampling frequency are shown in Table 1.

**Table 1:** The coefficients *w_i_* refitted for each spectral sampling frequency using linear regression with simulation data.

Spectral Sampling Frequency (cm^−1^)	*w*_0_	*w*_1_	*w*_2_	*w*_3_	*w*_4_	*w*_5_	*w*_6_	*w*_7_	*w*_8_	*w*_9_	*w*_10_
0.5	−0.435	0.688	0.025	0.788	0.877	−0.535	−0.516	−0.795	0.575	−0.609	0.504
1	0.676	0.614	0.132	1.126	1.050	−0.693	−0.624	−1.170	0.586	−0.603	0.579
2	−0.372	0.738	−0.148	2.249	0.939	−2.158	−1.210	−1.339	0.953	0.259	0.720
4	−0.080	0.531	1.555	−0.102	3.360	−3.164	−1.772	0.197	0.053	0.089	0.257
8	1.083	2.833	0.931	−1.686	2.256	−1.431	−2.140	1.996	0.683	−2.549	0.102

**Figure 8:**
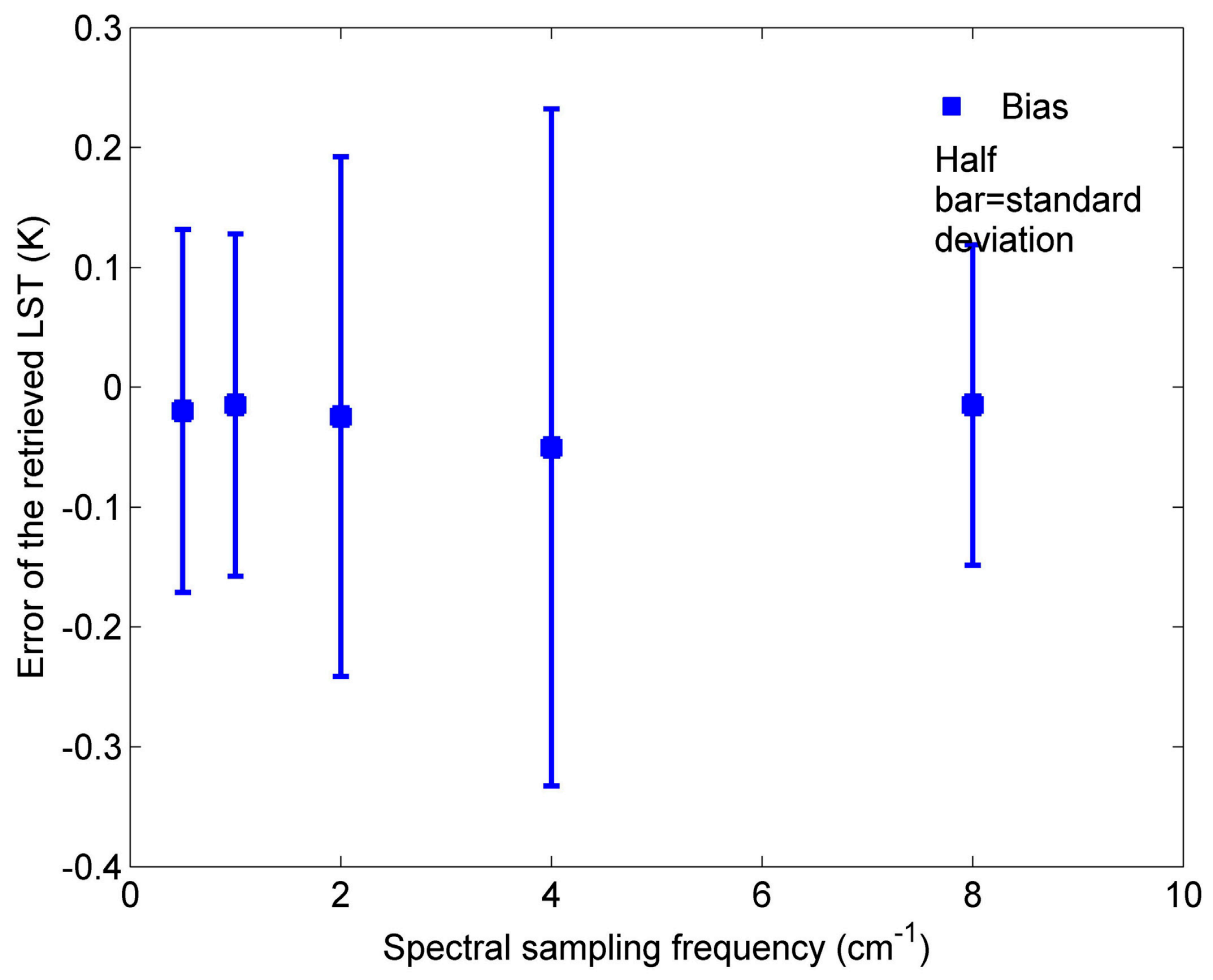
Errors of the ST retrieved using Equation (3) with refitted coefficients *w_i_* from each of the five simulation databases with 39 atmospheric profiles as a function of spectral sampling frequency.

To evaluate the accuracy of the ST retrieved using refitted coefficients *w_i_*, an independent simulation database for each spectral sampling frequency is resampled from the simulation data mentioned in Section 3 using each ISRF. The refitted coefficients *w_i_* and the 10 centre wavenumbers determined in Section 2 are then used for retrieving ST from these five independent simulation databases. The ST errors that were retrieved using Equation (3), with the refitted coefficients from each of the five independent simulation databases, are analysed with the spectral sampling frequencies and shown in Figure 8.

The coefficients *w_i_* refitted for each spectral sampling frequency using simulation data vary significantly with spectral sampling frequencies. Therefore, the coefficients in Equation (3) are dependent on spectral sampling frequency. The biases of the ST retrieved by Equation (3) with refitted coefficients *w_i_* from each of the five independent simulation databases vary between −0.01 K and −0.05 K, and the corresponding standard errors of the retrieved ST for each independent simulation database are less than 0.30 K. The ST can be retrieved accurately using Equation (3) with the refitted coefficients *w_i_* (*i* = [0,p]) for each spectral sampling frequency.

### 4.2. Sensitivity to Instrumental Noise

**Figure 9:**
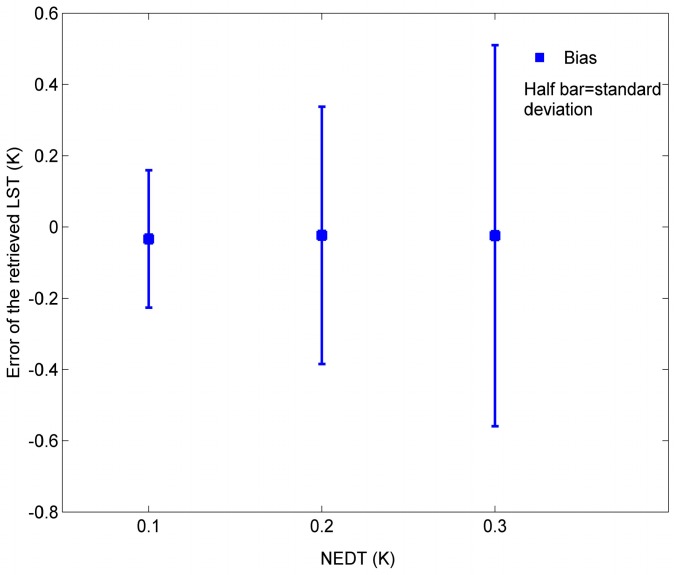
Errors of the ST retrieved using Equation (3) from each the three simulation databases as a function of instrumental noise.

To do this sensitivity analysis, three simulation databases are created by adding to noiseless IASI data noises with NE∆T = 0.1 K, 0.2 K, and 0.3 K, respectively. The noiseless IASI data is created using simulation data with 39 atmospheric profiles, as mentioned in Section 3. For each noiseless IASI spectrum, 20 noise-added IASI spectrums are created for each level of noise, including 20 random noises with the dimension of 1601. Each random noise is generated by the Matlab random number generator with corresponding NE∆T. The centre wavenumbers at channel *i* and coefficients *w*_i_, used for retrieving ST from the three simulation databases above, are those determined in Section 2. Figure 9 depicts the errors of the ST retrieved using Equation (3) from each of the three simulation databases as a function of instrumental noise.

With NE∆T instrumental noise growing from 0.1 K to 0.3 K, the standard error of the retrieved ST goes from 0.19 K to 0.53 K. Therefore, the impact of instrumental noise on the accuracy of the ST retrieved by Equation (3) is of the order of magnitude of the instrumental noise.

## 5. Application to Satellite Data

**Figure 10:**
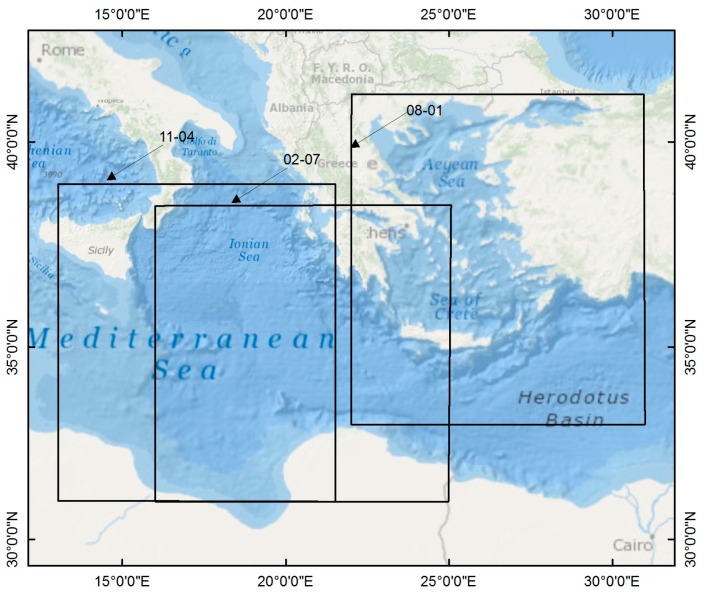
Target area used for comparing the ST retrieved by our method from Metop-A IASI data with Metop-A AVHRR SST product.

Our developed method is applied to Metop-A IASI data observed over the Mediterranean Sea. The Mediterranean Sea is in the region with a latitude of 30° N–43° N and a longitude of 12° E–32° E shown in Figure 10.

### 5.1. Data for This Application

The L1c image of Metop-A IASI has 8461 thermal infrared channels in the spectral interval of 645–2760 cm^−1^ (3.6–15.5 μm) with a spectral sampling frequency of 0.25 cm^−1^. The spatial resolution of IASI image at nadir point is 12 km. The scan angle at the end of each scan line is 48.98°. IASI on Metop-A scans the Mediterranean area in mid-morning orbit every day. The five-minute Metop-A IASI images sensed in the morning on three days in the year 2014 (February 2, August 1 and November 4) are collected for this study.

The Advanced Very High Resolution Radiometer (AVHRR) on the same satellite has two thermal infrared channels centred at 10.8 μm and 12 μm. The level 2 sea surface temperature (SST) product form AVHRR with a spatial resolution of 1 km is used to validate the SST retrieved by our developed method. The SST product from AVHRR is retrieved using the split-window method. The algorithm for deriving this SST product can be seen in [45].

### 5.2. Application to the Mediterranean Sea

Our developed method is applied to part of the collected IASI data, which has a viewing zenith angle less than 15° (the surface area covered by this selected data is shown in Figure 10 with rectangles). The cloud information in the AVHRR SST product is used to select the clear-sky IASI data. Only IASI data with more than 90% clear AVHRR pixels is used for this evaluation. In total, there are 386 matched IASI samples used for this application.

**Figure 11:**
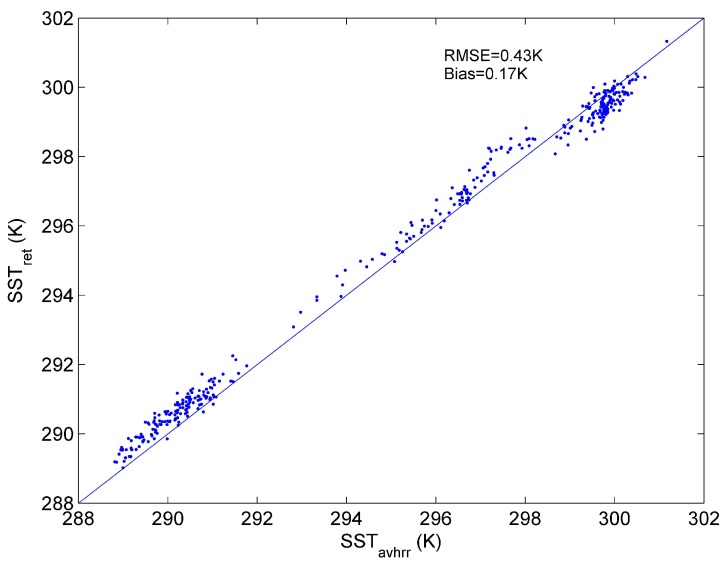
Comparison of the ST retrieved by our method from Metop-A IASI data with Metop-A AVHRR SST product over the Mediterranean Sea on three typical days. (ST_ret_ = the retrieved ST, SST_avhrr_ = the AVHRR SST).

The comparison of the ST retrieved by our developed method from IASI with the SST product from AVHRR is shown in Figure 11. From this figure, we can see that the RMSE of the ST from IASI is 0.43 K. The ST of high-emissivity surfaces can be retrieved accurately from satellite data by our method.

**Figure 12:**
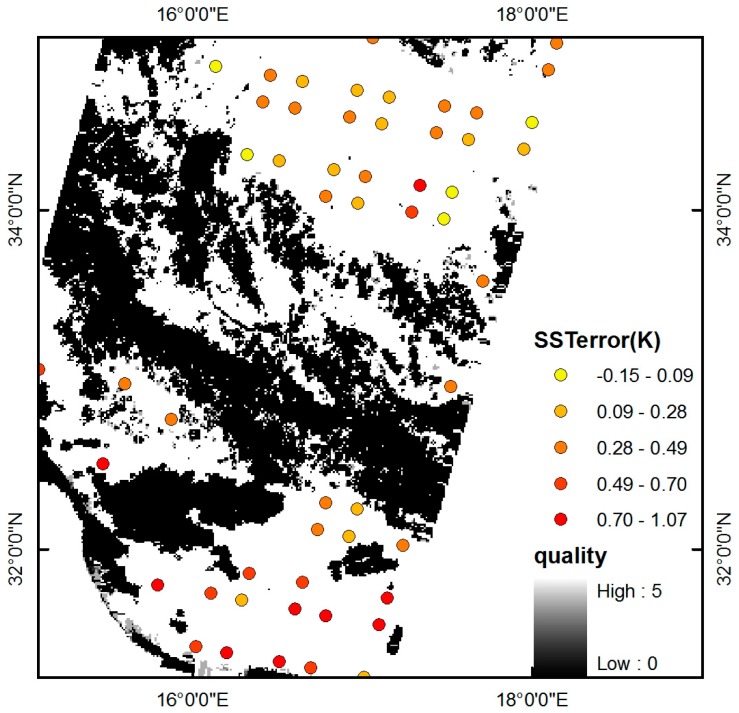
Error of the retrieved ST (IASI-AVHRR) plotted on a quality image of Metop-A AVHRR SST product over a typical part of the Mediterranean Sea on 4 November 2014. (SSTerror = SST_iasi_ − SST_avhrr_).

The spatial pattern of the ST error retrieved from the IASI image sensed on 4 November 2014 over part of the Mediterranean Sea is shown in Figure 12. The error of the retrieved ST is homogeneously distributed, and no important deviation is seen.

## 6. Conclusions

With the assumption that LSE is equal to unity, we have developed a multi-channel ST retrieval method for high-emissivity surfaces based on 10 HypTIR measurements of a radiometer with a spectral interval of 800–1200 cm^−1^ and a spectral sampling frequency of 0.25 cm^−1^. We have evaluated the method using independent simulation data. Moreover, we have analysed the sensitivity of the method to spectral sampling frequency and instrumental noise. This work draws the following conclusions:

(1) ST can be retrieved by our method from independent simulation data with RMSE of 0.21 K, using only 10 HypTIR measurements. This method is very accurate and promising.
(2) The coefficients *w_i_* of the method are dependent on a spectral sampling frequency. Nevertheless, ST of high-emissivity surfaces can still be retrieved accurately when the coefficients are refitted for each spectral sampling frequency.
(3) The impact of instrumental noise is not significant: the accuracy of the retrieved ST is of the order of magnitude of the instrumental noise.
(4) In comparison with the AVHRR SST product, ST of high-emissivity surfaces can be retrieved from satellite data with a RMSE of 0.43 K. The performance of our method is good for retrieving ST for high-emissivity surfaces from satellite data.

The drawback of our method is that it requires the assumption of LSE of unity. Our method can’t be applied to natural land surfaces yet. In future work, we will extend this method to natural land surfaces.
